# Characterization of green synthesized silver nanoparticles from *Falconeria insignis* Royle and its antimicrobial activity

**DOI:** 10.1371/journal.pone.0352435

**Published:** 2026-06-24

**Authors:** M. S. Amithalal, Mari Sumayli, E. S. Abhilash, Yehia Hazzazi, Ugur Azizoglu, Sajjad Sajjad, A. El-Shabasy

**Affiliations:** 1 P.G. Department of Biology & Botany, Sree Narayana Guru College, Chelannur, Kerala, India; 2 Department of Biology, College of Science, Jazan University, Jazan, Saudi Arabia; 3 Department of Crop and Animal Production, Safiye Cikrikcioglu Vocational College, Kayseri University, Kayseri, Turkey; 4 Genome and Stem Cell Center (GENKOK), Erciyes University, Kayseri, Turkey; 5 Department of Biotechnology and Genetic Engineering Hazara University Mansehra, Mansehra, Pakistan; Srimad Andavan Arts and Science College, INDIA

## Abstract

*Falconeria insignis* Royle leaf extract was utilized for the first time as a natural reducing and stabilizing agent in a green precipitation method to synthesize silver nanoparticles devoid of hazardous chemicals. A change in hue from light yellow to pale brown showed that AgNPs were formed. This was confirmed by UV-visible spectroscopy, which showed a unique surface plasmon resonance peak at 300–350 nm. The FTIR investigation found hydroxyl, carbonyl, amide, C–N, and C–O functional groups. This showed that proteins, phenolics, and flavonoids can bio-reduce and cap. XRD confirmed that the silver was nanocrystalline and had a mean crystallite size of 19 nm. The SEM showed predominantly non-spherical and heterogeneous in overall shape. Biogenic AgNPs are different from crude extract and silver nitrate, had much higher antibacterial activity against *Staphylococcus aureus*. ANOVA verified statistically significant differences between treatments. According to these findings, *F. insignis* Royle is a viable, sustainable bioresource for the manufacture of green AgNP with possible biomedical antibacterial uses.

## Introduction

Nanotechnology is a rapidly developing field, which focuses on designing, manufacturing and using materials in the nanometre scale, which is typically in the range of 1–100 nanometres. This field is an interdisciplinary branch of science, combining several other sciences, including chemistry, biology, materials science, and engineering, to develop instruments and systems that are more functional. Because of its interdisciplinary character, nanotechnology enables scientists to manipulate matter at the molecular scale, leading to the paradigm shift in medicine, electronics, energy and environmental science.Such materials are currently under research in such fields as medicine (Amedome Min-Dianeyet al., 2018) [1], agriculture (Rastogi et al., 2019) [2], environmental science (Singh and Verma, 2018) [3], electronics (Tamilvanan et al., 2014) [4], and energy storage (Manj et al., 2018) [5].

Silver nanoparticles (AgNPs) are widely studied metallic nanoparticles and used in particularly in biomedical sciences. Their properties include higher surface-area-to-volume ratio, plasmonics properties, as well as strong antibacterial action make them highly effective in diverse applications including diagnostics, therapies etc. (Rai et al., 2012 [6]; Franci et al., 2015 [7]). Unlike other such noble metals as gold or platinum, the silver nanoparticles can offer a unique combination of low cost and high levels of biological activity, which makes them particularly attractive in medical applications (Zhang et al., 2016) [8].

AgNPs are considered to be the most popular among all metallic nanoparticles due to the characteristics of physical and chemical properties, different applications in biomedicine, electronics, and catalysis (Abbas et al., 2024) [9]. Silver nanoparticles (AgNPs) play an extremely important role in nanomedicine in the diagnostics and treatment. Their features such as dimensions, morphology, surface area and distribution play a vital role in determining their behaviour and toxicity. Different methods are used to characterise such particles such as UV-Vis spectroscopy, X-ray diffraction (XRD), Fourier-transform infrared spectroscopy (FTIR), scanning electron microscopy (SEM) etc. The shift to a green method has increased the concern of the green manufacturing of nanoparticles. The process employs the use of plant extracts, which have high levels of physiologically active chemicals that facilitate the decrease of metal ions and stabilisation of nanoparticles. The synthesis factors such as size, shape, and the relationship between the syntheses and the environment are important to optimise the properties necessary.

AgNPs have been used widely due to their outstanding physicochemical properties which are primarily the antimicrobial properties. Among their most notable applications are in medicine where theAgNPs have been used in the dressing of wounds, surgical masks and medical device coatings to prevent microbial infections. They have a wide-spectrum antimicrobial activity, which disrupts the cell membrane, produces the reactive oxygen species, and binds to bacterial DNA and prevents replication and cell activities (Rai et al., 2009 [10]; Morones et al., 2005 [11]).

### A natural agent to green synthesis: *Falconeria insignis* royle

The fact that plant-derived agents are biocompatible and biodegradable is encouraging because it allows them to replace common chemical reagents in the production of nanomaterials (Iravani et al., 2014 [12]). *Falconeria insignis* Royle is a species that belongs to the Euphorbiaceae family and is native to dry forests and rocky areas in India. It is a shrub or small tree up to 6 meters tall that generates a milky latex which is used to treat skin disorders and also inflammation traditionally.

By taking advantage of the two-fold advantages of medicinal efficacy and environmental sustainability, the incorporation of *F. insignis* Royle in nanoparticle production systems would help in the evolution of new antimicrobial agents that can be used in clinics and industry.

*F. insignis* Royle, in this regard, is an unexploited but promising candidate to the green synthesis of silver nanoparticles (AgNPs), particularly since it has a rich arsenal of reducing and capping agents in its naturally occurring leaf and stem extracts. Although the plant has undergone preliminary studies in the area of phytochemical and antimicrobial, its opportunities in nanotechnology usage are still untapped, which gives a new orientation to the research.

A wide variety of bioactive compounds like alkaloids, flavonoids, terpenoids, and phenolics are present in this plant with antimicrobial and anti-inflammatory properties. These phytochemicals assist in reducing and stabilizing silver ions and thus allow the production of silver nanoparticles by the green method. These compounds have been characterized by antioxidant, antimicrobial and anti-inflammatory properties, thus the plant has become a potential candidate in the synthesis of metal nanoparticles using green synthesis. It has been shown that these biologically derived nanoparticles showed increased antimicrobial activity, which is probably explained by a synergistic approach to silver and plant-based bioactives (Patil et al., 2023) [13]. Although the green synthesis of silver nanoparticles using plant extracts is not new, the given work uses *Falconeria insignis* Royle as a new bioresource in the production of nanoparticles. *F. insignis* is a medicinal plant which thrives in some ecological niches and contains a large number of diverse phytochemicals, e.g., alkaloids, flavonoids, and latex-associated bioactives (Yadav et al., 2025) [14] Therefore, the use of *F. insignis* Royle in the synthesis of nanoparticles fits this objective in green chemistry in addition to aiding in the identification of new antimicrobial agents.

### Materials and methods

## Collection and preparation of plant material

Plant material: *Falconeria insignis* Royle

**Fig 1:**
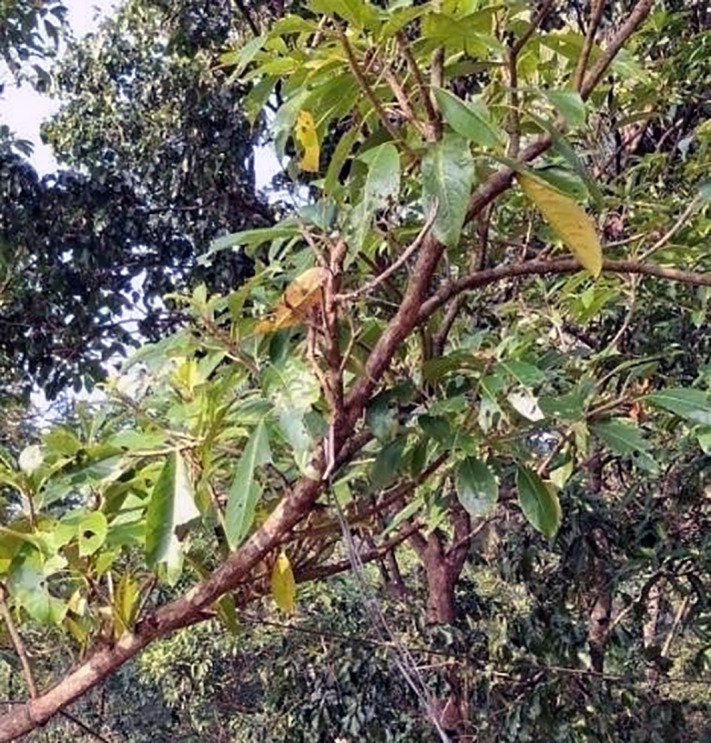
*Falconeria insignis* Royle plant habit.

*Falconeria insignis* Royle (Fig 1) fresh, healthy leaves were collected at Chelannur (The geographical coordinates of the site of plant collection is 11°20’30"N latitude and 75°48’30"E longitude). There were no particular permissions needed in these places for collection since it is the college campus of the first author were students and teachers are free to collect plant specimens for their studies. The global IUCN assessment has not listed *Falconeria insignis* Royle as endangered. It is also ensured that the field studies were not involved in the collection of endangered or protected species. 20 grams clean cut leaves were gently crushed and dissolved in 100 mL of distilled water. The mixture was placed at 60° C and stirred repeatedly (1 hour) so as to aid in the extraction of bioactive elements. The mixture was heated down to room temperature after which it was filtered through Whatman No.1 filter paper. The filtrate was subsequently collected and it was to be used as the plant extract in the synthesis of nanoparticles.

### Silver nanoparticles synthesis

Freshly made aqueous solution of 0.1 M silver nitrate (AgNO3) was used in the synthesis of the silver nanoparticles(AgNPs). 5 mL of *Falconeria insignis* Royle leaf extract was added dropwise into 45 mL of the 0.1 M AgNO3 solution at ambient conditions (Jeeva et al., 2014) [15].

### Fourier- Transform Infrared Spectroscopy (FTIR)

To separate the particles for FTIR measurement, the silver nanoparticles were spun at 10,000 rpm for 30 minutes. Deionised water was added three times to the pellet to get rid of any free proteins and phytochemicals. After the vacuum-dried pellet, FTIR spectroscopy was used to find the functional groups that use the plant extract to reduce and stabilise the nanoparticles (Das et al., 2010) [16].

### Scanning Electron Microscopy (SEM)

A colloidal suspension of AgNP was put on a clean glass slide in the form of a drop, and it was left to dry with no obstruction. A sputter coater was then used to cover the dried sample with a thin layer of gold in order to increase conductivity and minimize the effects of charging during imaging. It was followed by the coated sample being viewed under a SEM (Model: JEOL JSM-6390) at accelerating voltage of 10–20 kV. The SEM images will give specific information on the morphology of the surface, size, and shape of the nanoparticles that are synthesized (Das et al.,2010) [16].

### .X-Ray diffraction

X-ray diffraction analysis was conducted to compute the crystallites size of the nanoparticles. The Debye Scherrer equation was used for calculation: D = Kl / (b cos θ) Where: D is the average size of crystallites, K is the shape factor (0.89), l is the wavelength of X-rays, b is the full width at half maximum (FWHM) of the diffraction peak (in radians) and θ is the Bragg angle (Das et al., 2010) [16].

### Antibacterial activity

Kirby- Bauer disc diffusion method was used to determine the antimicrobial activity of the synthesized silver nanoparticles as stated by Pal et al. (2007) [17]. The details of the antimicrobial methodology of analyzing the efficacy of silver nanoparticles (AgNPs) by use of the Kirby-Bauer Disk Diffusion method are as follows.

1. **Bacterial Inoculum Preparation**: The antimicrobial effect of the prepared AgNPs was to be evaluated against clinical pathogen; *Staphylococcus aureus* (Gram-positive). Bacterial strains were grown in the Nutrient Broth and incubated at 37 ° C to reach the log growth stage of the bacterial strains. The turbidity of the cultures was brought to 0.5 McFarland standard (about 1.5 x 108 CFU/mL) to attain a uniform concentration of bacteria.
2. **Preparation of sterile paper discs**: Antimicrobial susceptibility filters using sterile, blank, paper discs (usually 6 mm in diameter) were prepared as the vehicle of delivery of the testing agents. The following treatment groups were impregnated in these discs with the following volumes. AgNPs and AgNO 3 concentrations were adjusted to a normalized value of mg/mL.

AgNPs Solution: Biosynthesized silver nanoparticles in *Falconera insignis* (1 mg/mL)*.*
Crude Leaf Extract: Aqueous extract of *F. insignis* (1 mg/mL)*.*
Silver Nitrate (Ag NO_3_): (1 mg/mL) (precursor control).

Positive Control: The comparative efficacy was done with the help of the positive control which were Standard antibiotic discs (Azithromycin 15 μg).

1. **Inoculation:** 100 μL of the bacterial suspension was placed in the middle of the sterile Mueller-Hinton Agar (MHA) plates using a sterile L-shaped glass spreader to form a confluent growth on the plates.
2. **Placement of Disc:** The impregnated paper discs were put on the surface of the inoculated agar plates with the help of sterile forceps with ensuring sharp contact to enable the diffusion of the agents into the medium.
3. **Incubation:** The plates were incubated at 37 °C during 24 hours in an upright position. Measurement and Statistical Analysis. After incubation, the plates were inspected regarding the Zones of Inhibition (clear areas around the discs in which bacterial growth was inhibited). A metric ruler was used to measure the diameter of these zones in millimeters (mm). All the experiments were conducted in triplicates in order to be reliable. To show the significant difference between the treatment groups, the results were presented in the form of Mean ± Standard Deviation (SD) and statistically compared with the help of One-Way ANOVA (P < 0.05).

## Results and discussion

When AgNO_3_ solution was added to the *Falconeria insignis* Royle leaf extract, the colour changed from light yellow to pale yellowish brown and this meant that silver nanoparticles (AgNPs) had been formed. This colour alteration may be explained by the facts that the surface plasmon resonances (SPR) of silver particles were excited where the conduction electrons on the surface of the nanoparticles are vibrating at the same frequency as the incident light wave (Mulvaney, 1996 [18]; Yusuf, 2017 [19]).These phytochemicals present in leaf extract not only reduce Ag + ions to metallic Ag0, but also close the nanoparticles preventing agglomeration (Song and Kim, 2009 [20]; Iravani, 2014 [12]). Similar colour changes in other biomolecules syntheses by other plants have also been reported which further enhances the use of plant-derived biomolecules in the nanoparticles (Veerasamy et al., 2011 [21]). The use of the *Falconeria insignis* Royle with latex-based proteins as natural capping agents provided superior stability even after a month without any visible change compared to standard aqueous leaf extracts. The similar results were obtained by Bar et al., 2009 [22].

### UV-visible absorbance spectroscopy

This absorption pattern indicates that AgNPs were successfully synthesized because the silver nanoparticles exhibit SPR bands at 300–450 nm. In this region, the absorbance was high and this shows the presence of the colloidal silver nanoparticles. This spectral evidence supports the use of *Falconeria insignis* Royle extract as reducing and stabilizing agent in the synthesis that the green synthesis process was successful.

**Fig 2:**
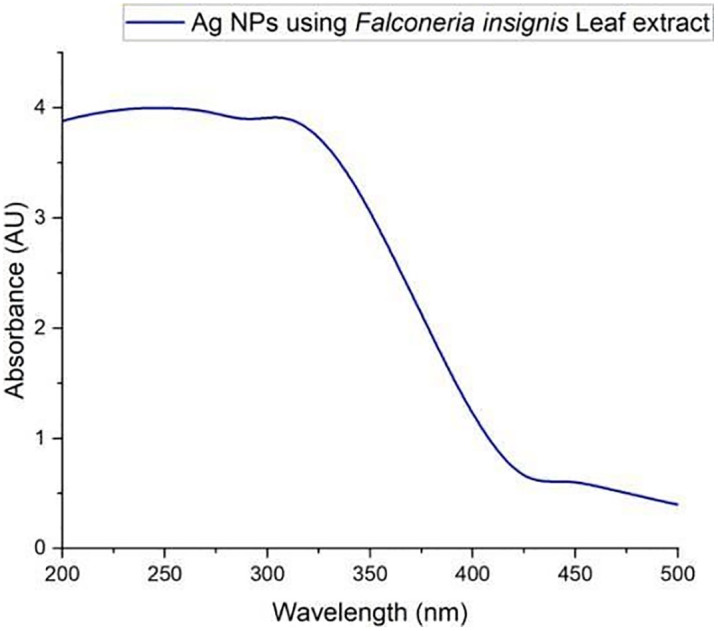
UV Spectrometry of AgNPs.

The success of silver nanoparticles synthesis is proven by the presence of the SPR peak that is unique (around 380 nm) in the UV-Vis spectrum because the success is expected to be 370-450 nm that is commonly ascribed to the spherical or quasi-spherical AgNPs (Mulvaney, 1996 [18]; Veerasamy et al., 2011 [21]). The absence of the peak higher than 450 nm also indicates that the nanoparticles are not highly aggregated or form large as was also shown by Ahmed et al. (2016) [23] and Song and Kim (2009) [20]. Such findings also confirm the effectiveness of *Falconeria insignis* Royle as an effective bioreducing and stabilizing agent in synthesizing AgNPs greenly (Fig 2).

### Fourier–transform infrared spectroscopy (FTIR)

**Fig 3:**
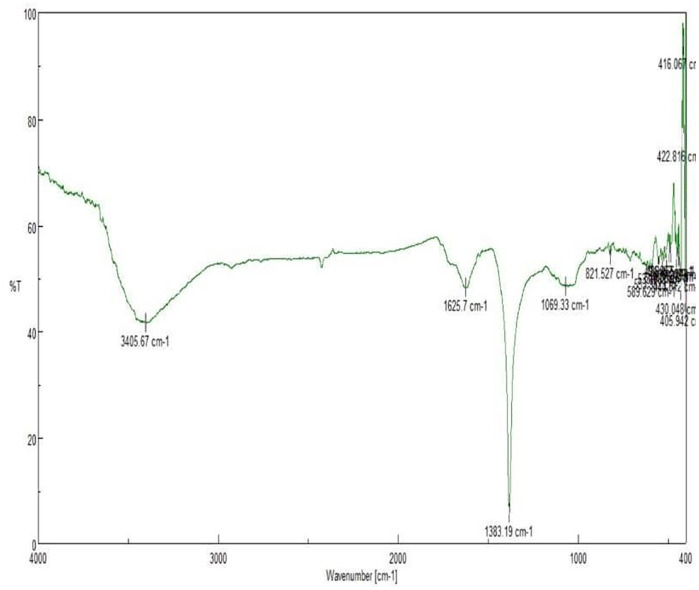
FTIR analysis of AgNPs.

There is a broad peak of 3405.67 cm-1 which signifies O-H stretching frequencies of hydroxyl groups that are usually present in alcohols and phenolic compounds. It has the highest peak of 1625.7 cm-1 which may be due to C=C stretch of aromatic rings or C=O stretch of amide groups in proteins implying the presence of flavonoids or proteins that may help capped and stabilize AgNPs. The high intensity at 1331.13 cm-1 represents the presence of C-N stretching vibrations that also substantiate the presence of amine containing biomolecules such as proteins in the nanoparticle synthesis process. It has a maximum at 1093.33 cm-1 indicating that there are C-O stretching vibrations indicating that there are alcohols, ethers, or esters in the leaf extract. The other observed peaks at 821.127 cm-1 and 416.046-422.187 cm-1 were found to be aromatic C-H bending vibration and metal-oxygen (Ag-O) vibration, respectively and this confirms the successful formation of silver nanoparticles (Fig 3).

The presence of the O-H stretching peak justifies the presence of polyphenols and flavonoids, which have high reducing powers, in the study as already indicated by Das et al.(2010) [16], Kaviya et al. (2011) [24] and Iravani (2014) [12]. Those amide bonds suggest that proteins are also natural capping agents like in the case of Song and Kim (2009) [20]. C-N and C-O band presence indicates the role of alkaloid, amino acid, and sugar in preserving the nanoparticles in the studies by Veerasamy et al. (2011) [21] and Ahmed et al. (2016) [23]. The fact that Ag-O associated peaks form in the lower wave numbers is a strong indication that silver nanoparticles have been successfully synthesized, as the FTIR profiles as explained by Jain and Mehata (2017). All these findings confirm the green synthesis capability of *Falconeria insignis* Royle as the resultant of active participation of biofunctional components.

### Scanning Electron Microscopy (SEM) analysis of silver nanoparticles

The green synthesised nano particles (AgNPs) are non-spherical and heterogeneous in shape and also strong agglomeration is observed, forming clustered structures.

Surface texture appears rough and granular, indicating its possible polycrystalline nature.

The sizes of the particles were determined to be varying and measured were found to be in the range of 18.50 nm to 20.01 nm.

**Fig 4:**
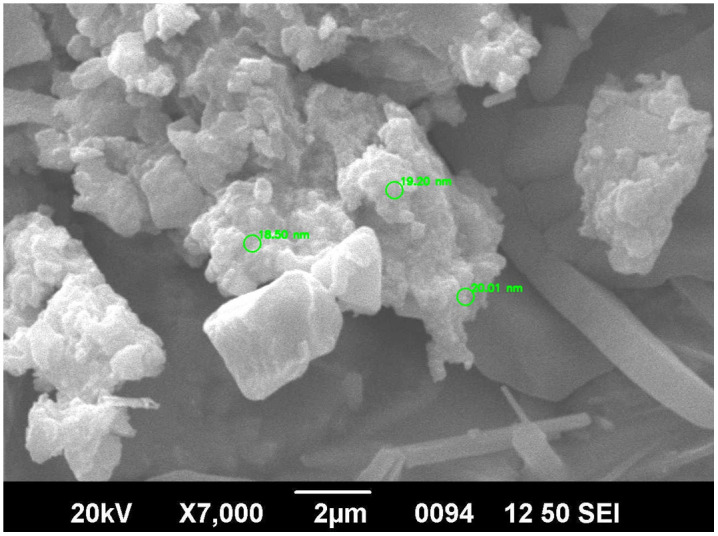
7,000x Magnified SEM image of AgNPs.

SEM micrograph supports the fact that silver nanoparticles are remarkably narrow size distribution for these nanoparticles. shape were biosynthesized successfully, indicating that the presence of certain phytochemicals in *Falconeria insignis* Royle could be the determinant of nanoparticles during the biosynthesis pathway. It aligns to most of the reports about the green synthesis, most of them usually produce spherical particles (Veerasamy et al., 2011 [21]; Ahmed et al., 2016 [23]. Other few studies have also reported similar non spherical morphologies, including the cases of *Aloe vera* and *Terminalia arjuna* extracts, in which directional binding of proteins or flavonoids was the cause of the development of anisotropic growth (Jain and Mehata, 2017) [25] (Fig 4). Although the general size of nano particles in *Falconeria insignis* Royle (18.50 nm to 20.01 nm) is small, the non-spherical and heterogeneous shapes also vary, which also confirms anisotropic growth. It aligns with earlier studies (Xia & Halas, 2005) [26]. Even small variations in the synthesis conditions or the availability of different directing agents may cause a wide range of anisotropic forms in a population. The textured surface of rough and granular texture, which is suggestive of polycrystalline structure, is also in line with anisotropic growth. Anisotropic growth can be the preferential addition of atoms to specific crystal faces. Growth may occur differently on the different surfaces of the grains in polycrystalline materials, which can add to the rough texture observed, as well as affect the ultimate non-spherical morphology (Jana et al., 2001) [27].

### X-ray diffraction

**Fig 5:**
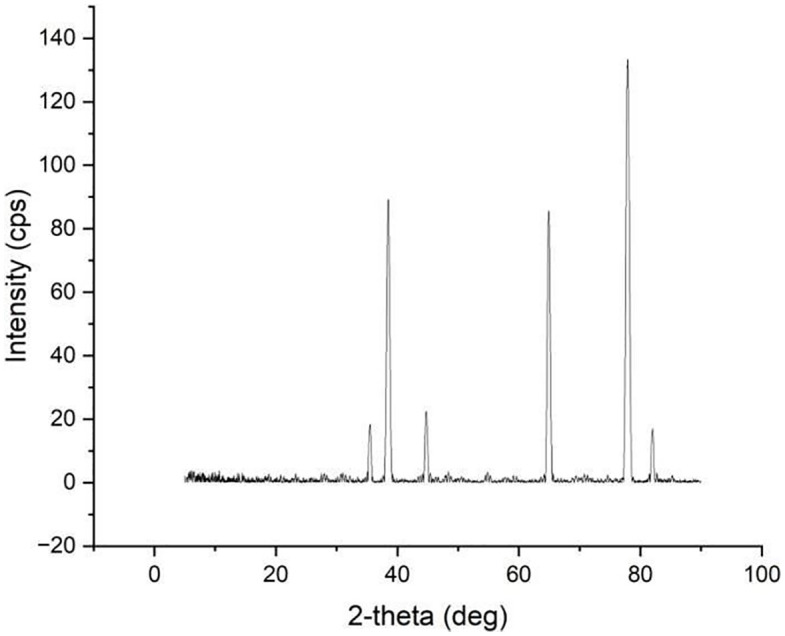
XRD Analysis of AgNPs.

The high order diffraction peaks were found at the values of 35.51deg, 38.50deg, 44.76deg, 64.94deg, 77.92deg, 82.00deg respectively, the characteristic reflections of the (111), (200), (220), (311), (222), and (400) crystallographic planes respectively. Debye-Scherrer equation was applied to estimate the crystallite size. The observed sizes of the crystallites calculated for the observed peaks were between 14.84 nm and 25.55 nm. Mean crystal size was calculated to be around 19.10 nm aligns with the SEM calculated values which implies that the nanoparticles synthesized are in the nanometer range and they are nanocrystalline in nature (Fig 5).

The observed characteristic Bragg reflections at 38.50 deg, 44.76 deg, and 64.94 deg are consistent with the standard face-centered cubic (FCC) structure of silver nanoparticles as it is found in the literature (JCPDS card no. 04-0783) (Zhou et al., 2011 [28]; Tamilvanan et al., 2021 [4]) (Table 1).

**Table 1:** Crystalline size of silver nanoparticles.

Peak position theta	FWHM	Crystallite size D(nm)	Average crystallite size D (nm)
35.51	0.39593	21.06827811	19.10
38.50	0.56697	14.84158696	
44.76	0.46248	18.57663218	
64.94	0.52422	17.96264835	
77.92	0.61572	16.59250327	
82.00	0.41194	25.55231216	

### Anti-bacterial activity of silver nanoparticles from *Falconeria insignis*

**Fig 6:**
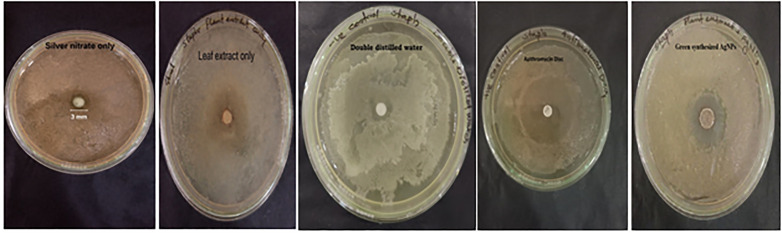
Antibacterial activity against *Staphylococcus aureus* (Gram positive).

The antimicrobial disc has found to be more inhibition 28.33 mm (Fig 6), followed Silver nano particle 21 mm (Fig 6), leaf extract alone 17 mm, Double distilled water 0 mm, Silver Nitrate alone showed 3 mm bacterial inhibition in the case of *Staphylococcus aureus* (Gram positive) (Table 2).

**Table 2:** Inhibitory zone of disc diffusion assay *Staphylococcus aureus* (Gram positive).

Samples (1mM)	Inhibitory zone diameter (mm)
Leaf Extract	17 ± 1
Leaf extract with Silver Nano Particle	21 ± 1
Silver Nitrate	3 ± 1
Antimicrobial DiscAzithromycin (15 µg)	28.33 ± 1.53

The findings of the ANOVA showed that there is a statistically significant difference among treatment groups (F = 231.25, p = < 0.0001), which confirms that the antimicrobial activities of the samples differed significantly (Table 3). One-way ANOVA showed that there is a significant difference between the treatment groups (F = 3,8 = 231.25, p = 0.0001), which means that the identified changes are statistically significant at the confidence level of at least 99.99. Of the samples, azithromycin had the higher zone of inhibition of 28.33 mm, whereas the plant leaf extract itself had the least zone (21 mm). The extract prepared by nanoparticles had higher activity compared to the silver nitrate alone and the crude extract implying greater antimicrobial potential owing to nanoparticle additions (Table 4).

**Table 3:** One-Way ANOVA performed for the four treatments pertaining to *Staphylococcus aureus.*

Source	Sum of Squares	df	F-value	p-value
**Between Groups**	1007.86	3	231.25	< 0.0001
**Within Groups**	11.62	8	—	—
**Total**	1019.48	11	—	—

Descriptive statistics (n = 3 per group). (Four treatments, assumed n = 3 replicates per treatment based on the reported mean ± SD).

**Table 4:** Post-hoc comparisons (Tukey HSD, α = 0.05).

Comparison (A vs B)	Mean difference (A − B)	Adjusted p	95% CI for difference	Significant?
Azithromycin vs Leaf + AgNP	+7.33 mm (Azithromycin larger)	0.0002	4.31 to 10.35 mm	Yes
Azithromycin vs Leaf Extract	+11.33 mm	<0.0001	8.31 to 14.35 mm	Yes
Azithromycin vs Silver Nitrate	+25.33 mm	<0.0001	22.31 to 28.35 mm	Yes
Leaf + AgNP vs Leaf Extract	+4.00 mm (AgNP larger)	0.0121	0.98 to 7.02 mm	Yes
Leaf + AgNP vs Silver Nitrate	+18.00 mm	<0.0001	14.98 to 21.02 mm	Yes
Leaf Extract vs Silver Nitrate	+14.00 mm	<0.0001	10.98 to 17.02 mm	Yes

### Rank order of activity (largest → smallest zone)

Azithromycin (28.33 ± 1.53 mm)> Leaf + AgNP (21 ± 1 mm)> Leaf Extract (17 ± 1 mm)> Silver Nitrate (3 ± 1 mm). No comparison with zero is within the confidence intervals, which proves that there is no overlap between groups. Adding AgNPs to the leaf extract boosts the inhibition zone by 4.0 mm (95% CI 1.0–7.0 mm, p = 0.012). This induces the notion that green-synthesized AgNPs have a greater antimicrobial activity than the crude extract alone- a trend that has been repeatedly observed with plant-mediated AgNPs.

The results obtained in antibacterial activity indicate clearly that the biosynthesized silver nanoparticles (AgNPs) have strong inhibition circles than both *Falconeria insignis* Royle leaf extract and silver nitrate respectively (Fig 5). AgNPs generated more extensive areas of inhibition (21 mm for *Staphylococcus aureus*) (Table 2).It is found that leaf extract (17 mm) and green silver nano particles (21 mm), were superior to the negative control (Double distilled water) suggests better antimicrobial activity. It is possible to explain this enhanced activity of AgNPs by the smaller size of the particles, the greater ratio of the surface to the volume, and the capability to infiltrate into the bacterial cell wall and damage its structure and functions (Rai et al., 2012 [6]; Zazo et al., 2016 [29]; Yu et al., 2013 [30]).Tamilvanan et al. 2021 [4] received the same results as AgNPs synthesized using plant extracts were more active in antibacterial than crude extracts and silver salt solutions obtained using plant extracts. Furthermore, these phytochemicals of the *Falconeria insignis* Royle have probable capping activities that stabilize the nanoparticles and improve their contact with the microbial cells. The similar findings by Li et al. (2011) [31], who reported that the biologically synthesized AgNPs disrupt the bacterial membranes leading to cell lysis. In such way, the enhancing action of phytochemical and nanoscale silver seems to play an important role in the increased antimicrobial action of the study.

## Conclusion

This paper has managed to prove that silver nanoparticles (AgNPs) can be green synthesized by using *Falconera insignis* Royle leaf extract as a reducing and stabilizing agent. The synthesized AgNPs had a characteristic surface plasmon resonance peak at around 380 nm in UV-Vis spectroscopy, which confirmed the formation of AgNPs with a size of 14.84 to 25.55 nm (average 19.10 nm) by XRD and SEM analyses. FTIR spectra indicated that phytochemicals such as polyphenols, flavonoids, proteins, and amines were involved in capping the nanoparticles, which are known to stabilize the nanoparticles and give them their Non-spherical anisotropic morphology.

XRD patterns matched the face-centered cubic structure of silver (JCPDS 04-0783), with peaks at 38.50°, 44.76°, and 64.94° corresponding to (111), (200), and (220) planes. Non-spherical heterogeneous particles (18.50-20.01 nm) were observed in the SEM images with rough polycrystalline surfaces and agglomeration. These characteristics are in line with the stabilizing activity of the plant biomolecules that do not allow excessive aggregation and, therefore, there are no peaks above 450 nm in UV-Vis.

AgNPs exhibited better antibacterial effect against *Staphylococcus aureus* with a zone of inhibition of 21 mm, when compared to 17 mm with the leaf extract and 3 mm with silver nitrate. Statistical ANOVA has proved that there are significant differences between treatments (F = 231.25, p < 0.0001) and that the small size, high surface area, and bacterial membrane disruption capacity of the nanoparticles are the factors that contribute to the high performance. A p-value of < 0.0001 corresponds to a confidence level greater than 99.99%.

*Falconia insignis* Royle is a viable and sustainable biosynthesis of AgNP that is beneficial and superior to chemical biosynthesis in antimicrobial efficacies. Such results are helpful in general use in the fight against antibiotic-resistant bacteria. This paper is novel in that it is the first to report *F. insignis*-mediated synthesis of AgNPs and anisotropic, mostly unspherical nanoparticle morphology in an anisotropic, unlike the typically reported spherical particles in most plant-based systems.
